# Evaluation of omadacycline against intracellular *Mycobacterium abscessus* in an infection model in human macrophages

**DOI:** 10.1093/jacamr/dlad104

**Published:** 2023-09-15

**Authors:** S Jahanbakhsh, J Howland, M O Ndayishimiye Uwineza, M T Thwaites, C M Pillar, A W Serio, D M Anastasiou, D A Hufnagel

**Affiliations:** Microbiologics Antibiotic and Microbiome Research Center, Kalamazoo, MI, USA; Microbiologics Antibiotic and Microbiome Research Center, Kalamazoo, MI, USA; Microbiologics Antibiotic and Microbiome Research Center, Kalamazoo, MI, USA; Microbiologics Antibiotic and Microbiome Research Center, Kalamazoo, MI, USA; Microbiologics Antibiotic and Microbiome Research Center, Kalamazoo, MI, USA; Paratek Pharmaceuticals, Inc., King of Prussia, PA, USA; Paratek Pharmaceuticals, Inc., King of Prussia, PA, USA; Microbiologics Antibiotic and Microbiome Research Center, Kalamazoo, MI, USA

## Abstract

**Background:**

Omadacycline is an aminomethylcycline antibiotic in the tetracycline class that was approved by the US FDA in 2018 for the treatment of community-acquired bacterial pneumonia and acute bacterial skin and skin structure infections. It is available in both IV and oral formulations. Omadacycline has broad-spectrum *in vitro* activity and clinical efficacy against infections caused by Gram-positive and Gram-negative pathogens. Omadacycline is being evaluated in a 3 month placebo-controlled Phase 2 clinical trial of oral omadacycline versus placebo in adults with non-tuberculous mycobacteria (NTM) pulmonary disease caused by *Mycobacterium abscessus* (NCT04922554).

**Objectives:**

To determine if omadacycline has intracellular antimicrobial activity against NTM, bacteria that can cause chronic lung disease, in an *ex vivo* model of intracellular infection.

**Methods:**

Two strains of *M. abscessus* were used to infect THP-1 macrophages. Intracellular *M. abscessus* was then challenged with omadacycline and control antibiotics at multiples of the MIC over time to evaluate intracellular killing.

**Results:**

At 16 ×  the MIC at 72 h, omadacycline treatment of intracellular NTM yielded a log_10_ reduction in cfu of 1.1 (91.74% reduction in cfu) and 1.6 (97.65% reduction in cfu) consistent with killing observed with tigecycline, whereas amikacin and clarithromycin at 16 ×  the MIC did not show any reduction in cfu against the intracellular *M. abscessus*.

**Conclusions:**

Omadacycline displayed intracellular activity against *M. abscessus* within macrophages. The activity was similar to that of tigecycline; as expected, intracellular killing was not observed with clarithromycin and amikacin.

## Introduction

Non-tuberculous mycobacteria (NTM) are inherently antibiotic resistant, grow slowly, and have a complex intracellular lifestyle in the host, often evading the antibacterial effects of the phagolysosome by preventing maturation of the phagosome through neutralization of pH and prevention of host antimicrobial production.^1,2^ NTM are opportunistic pathogens, have known reservoirs in water systems of medical facilities, and often cause infections through injections of contaminated substances or through medical device implants.^3^*Mycobacterium abscessus*, one species of NTM, causes respiratory infections in patients with cystic fibrosis (CF), AIDS, COPD and other diseases in immunocompromised patients.^4,5^ Although NTM typically are not transmitted person-to-person, *M. abscessus* has been documented to spread between patients with CF.^6^

Omadacycline is a semi-synthetic derivative of tetracycline with indications of community-acquired bacterial pneumonia and acute bacterial skin and skin structure infections.^7^ Omadacycline has activity against both Gram-positive and Gram-negative organisms; not only does it have a tetracycline-unique ribosomal interaction, but it also retains activity against the vast majority of clinically relevant tetracycline-resistance mechanisms.^8^

*In vitro*, omadacycline displays activity against *M. abscessus* in broth microdilution and time–kill kinetics assays.^9,10^ Additionally, tigecycline, has shown activity against NTM in an intracellular killing assay targeting NTM in macrophages, whereas amikacin and clarithromycin did not show any intracellular activity.^11^ This study sought to determine whether omadacycline maintained antibacterial activity against intracellular *M. abscessus* in a differentiated human macrophage cell line, THP-1.

## Methods

### Test compounds

Omadacycline was provided by Paratek Pharmaceuticals (Lot No. CA20-0964). Micromyx provided the following comparators: tigecycline (USP; R09410), amikacin (Sigma; 058k0803) and clarithromycin (USP; G2I235), which were handled following CLSI guidelines.^12^ Testing ranges for omadacycline, tigecycline and clarithromycin were 0.03–32 mg/L, whereas for amikacin it was 0.06–64 mg/L.

### Test organisms

The test organisms evaluated were *M. abscessus* ATCC 19977 and MMX 9450, an isolate collected from a patient sputum sample in Indiana, USA in 2017. NTM were grown on Middlebrook 7H11 selective agar (Hardy Diagnostics; 137898, 14213) for 3–5 days at 30°C. They were then subcultured onto Middlebrook 7H11 non-selective agar (Hardy Diagnostics; 498795, 501472) and incubated for approximately 5 days at 30°C prior to use in the MIC assay. All test organisms were identified by a Bruker MALDI Biotyper (Bruker Daltonics).

### Broth microdilution MIC assay

MIC values were determined in duplicate using a broth microdilution procedure described by CLSI (M24, M62 and M100).^12–14^ The test medium used was CAMHB (BD; 1242967). All drug stocks evaluated were within CLSI published QC ranges against *Staphylococcus aureus* ATCC 29213 and *Mycobacterium peregrinum* ATCC 700686.

### Cell culture and intracellular antibacterial activity assay

Medium, conditions, treatment and recovery for intracellular killing assays were based on previous studies.^11,15^ THP-1 cells (ATCC-TIB-202; 70043382) were grown at 37°C with 5% CO_2_. To differentiate into macrophages, 1 mL of cell suspension (approximately 5 × 10^5^ cells/mL) was added per well in Roswell Park Memorial Institute culture medium (RPMI) with 10% FBS, 1% pen/strep and 200 nM phorbol 12-myristate 13-acetate (PMA; Sigma; MKCL1143) in a 24-well tissue culture plate containing sterile glass coverslips on the bottom of each well and incubated for 48 h. Following incubation, PMA medium was replaced with RPMI with 10% FBS without antibiotics and the cells were incubated for 3 days. Following incubation, the medium was removed from the wells with adherent macrophage, and 1 mL of RPMI with 2% FBS containing 5–7 × 10^5^ cfu/mL of a log-phase bacterial suspension at a multiplicity of infection (MOI) of 1:1 was added to the wells. Plates were then incubated for 6 h at 37°C with 5% CO_2_.

Medium was removed from each well prior to washing twice  in prewarmed Dulbecco’s phosphate-buffered saline (DPBS; Sigma; RNK9608). After washing, 1 mL of 200 µg/mL amikacin suspended in RPMI with 2% FBS was added to each well, then plates were incubated for an additional 2 h at 37°C with 5% CO_2_ to kill extracellular bacteria. The medium was removed prior to washing twice  in prewarmed PBS. After washing, 1 mL of 0×, 0.5×, 1×, 4× and 16×  the MIC of four antibiotics (omadacycline, tigecycline, amikacin and clarithromycin) suspended in RPMI with 2% FBS was added to the wells. At 0, 24, 48 and 72 h, coverslips were removed, blotted onto sterile cloth, and added to 50 mL conical tubes with 5 mL 1% (v/v) Triton X-100 (Sigma; 033K0605) in saline with 5 to 10 sterile 3 mm glass beads. Conical tubes were vortexed on high for 1 min to lyse the cells and release intracellular bacteria. Cell suspensions were serially diluted 1:10, plated on trypticase soy agar with 5% sheep blood, and agar plates were incubated at 35°C for approximately 72 h to allow for enumeration of viable bacteria.

### Statistical analysis

Changes in cfu/mL were compared by Student’s unpaired two-tailed *t*-test for time–kill assays. *P* values ≤0.05 were considered significant. All statistical analyses were performed using Microsoft Excel (Microsoft 365).

## Results

### Broth microdilution testing

The MIC values for omadacycline, tigecycline, amikacin and clarithromycin were 0.12, 0.12, 8 and 2 mg/L against *M. abscessus* ATCC 19977, respectively, and 0.5, 0.25, 8 and 2 mg/L against *M. abscessus* MMX 9450, respectively (Table S1, available as Supplementary data at *JAC-AMR* Online). Both strains were susceptible to amikacin and clarithromycin; there are no interpretive criteria established for omadacycline and tigecycline.^13^ These data were used to determine the drug exposures at multiples of the MIC described below.

### Intracellular activity of antimicrobials

**Figure 1.**
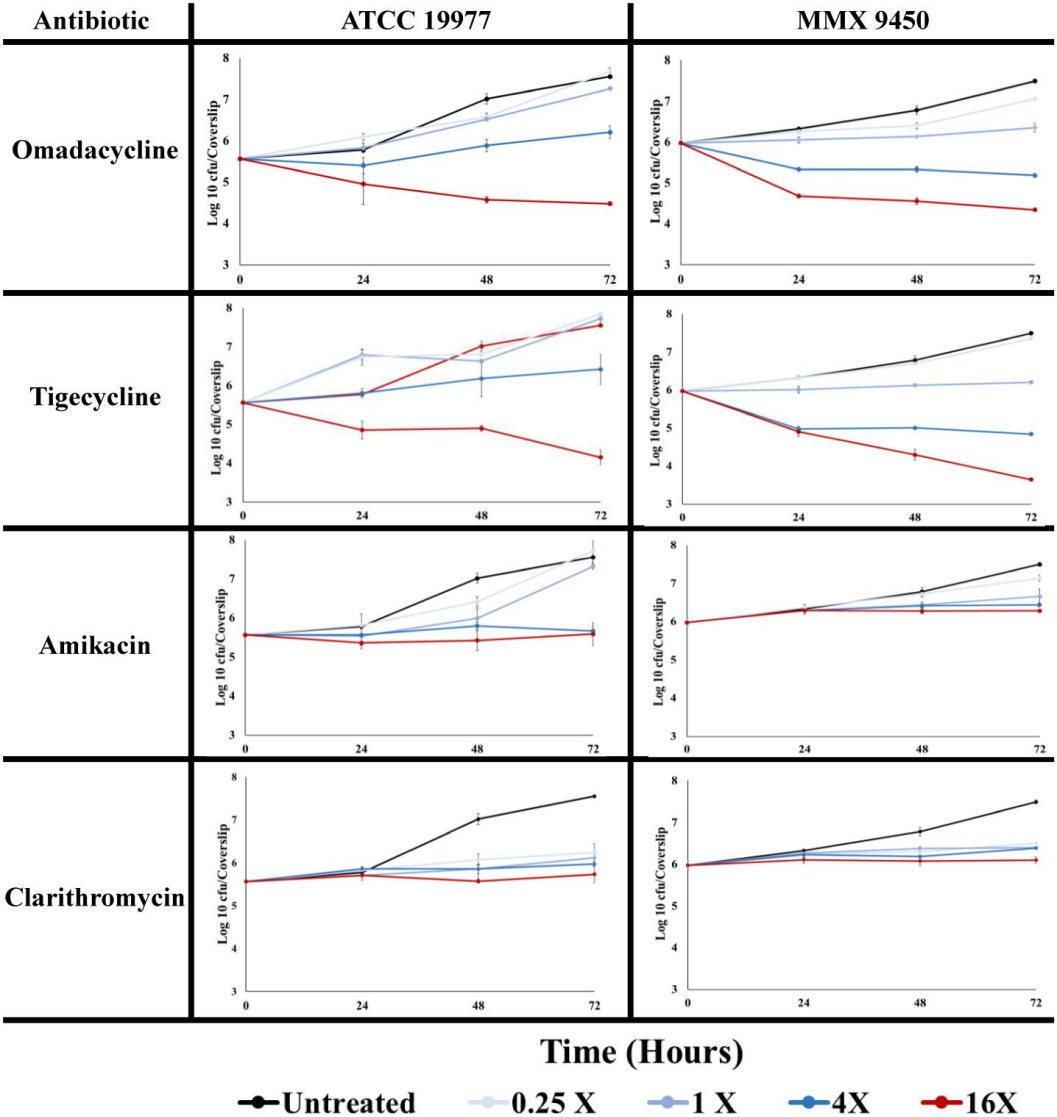
Intracellular activity of antibiotics over time against two *M. abscessus* strains in THP-1 macrophages. Graphs show the mean log_10_ cfu/coverslip of intracellular *M. abscessus* recovered at each timepoint in each condition during the intracellular killing assay. Error bars represents the SD of three independent wells.

In this study, four antibiotics at four concentrations were evaluated against two *M. abscessus* strains in triplicate. The change in mean log_10_ cfu/coverslip and time–kill kinetics for omadacycline, tigecycline, amikacin and clarithromycin compared with untreated controls at 0 h against *M. abscessus* ATCC 19977 and MMX 9450 are shown in Table 1 and Figure 1, respectively. Omadacycline at 16 × MIC demonstrated intracellular killing activity at 48 and 72 h, with log reductions of 1.0 (*P* value <0.001) and 1.1 (*P* value <0.001), respectively, for ATCC 19977, and log reductions of 1.4 (*P* value = 0.003) and 1.6 (*P* value = 0.002), respectively, for MMX 9450. Tigecycline at 16 ×  MIC at 48 and 72 h had log reductions of 0.7 (*P* value <0.001) and 1.4 (*P* value <0.001), respectively, against ATCC 19977, and log reductions of 1.7 (*P* value = 0.003) and 2.3 (*P* value = 0.002) when testing against MMX 9450. At 72 h and 16 ×  the MIC value against ATCC 19977 and MMX 9450, the percent reduction by omadacycline was 91.74% and 97.65% against the two isolates, whereas tigecycline showed percent reduction of 96.12% and 99.53%. Amikacin and clarithromycin did not exhibit any intracellular effect on either strain infecting THP-l macrophages (Table 1 and Figure 1).

**Table 1.** Change in cfu/coverslip of intracellular *M. abscessus* over time treated with omadacycline and comparators

Organism	Multiple of the MIC	Δ log_10_ cfu/coverslip relative to untreated control at 0 h											
		Omadacycline			Tigecycline			Amikacin			Clarithromycin		
		24 h	48 h	72 h	24 h	48 h	72 h	24 h	48 h	72 h	24 h	48 h	72 h
*M. abscessus* ATCC 19799	0.25×	0.5	1.0	2.1	1.2	1.1	2.2	0.3	0.9	2.2	0.3	0.5	0.7
	1×	0.3	1.0	1.7	1.2	1.2	2.3	0.0	0.4	1.8	0.1	0.3	0.6
	4×	−0.2	0.3	0.6	0.2	0.6	0.9	0.0	0.2	0.1	0.3	0.3	0.4
	16×	−0.6	**−1**.**0**	**−1**.**1**	−0.7	−0.7	**−1**.**4**	−0.2	−0.1	0.0	0.1	0.0	0.2
*M. abscessus* MMX 9450	0.25×	0.3	0.4	1.1	0.3	0.7	1.4	0.4	0.8	1.2	0.3	0.3	0.5
	1×	0.1	0.2	0.4	0.0	0.2	0.2	0.3	0.5	0.7	0.3	0.4	0.4
	4×	−0.6	−0.6	−0.8	**−1**.**0**	**−1**.**0**	**−1**.**1**	0.3	0.4	0.5	0.3	0.2	0.4
	16×	**−1**.**3**	**−1**.**4**	**−1**.**6**	**−1**.**1**	**−1**.**7**	**−2**.**3**	0.3	0.3	0.3	0.1	0.1	0.1

Bold text denotes  ≥ 1-log killing. Mean Δlog10 determined by the change in cfu/mL versus the starting intracellular cfu (0 h timepoint) from sampling triplicate wells of each condition. The difference in mean log_10_ cfu/coverslip recovered for two intracellular NTM isolates treated with multiples of the MIC of omadacycline, tigecycline, amikacin and clarithromycin over time versus the starting intracellular cfu.

## Discussion

There is a critical need for new antibiotics to treat diseases caused by *M. abscessus*, which is one of the most difficult-to-treat NTM species.^9^ Currently, few treatment options are available for *M. abscessus* MDR infections, and there is a lack of efficacy data against NTM in clinical trials, so new antibiotic therapeutics are urgently needed.^5,16^ However, most antibacterial studies for *M. abscessus* are conducted in *in vitro* models that do not account for the intracellular presence of *M. abscessus*. In this study, four antibiotics were evaluated in an *ex vivo* model of infection in a differentiated human macrophage cell line: omadacycline, tigecycline, amikacin and clarithromycin, for intracellular antimicrobial activity against two strains of *M. abscessus*.

Our data showed that both omadacycline (2 mg/L for ATCC 19977 and 8 mg/L for MMX 9450) and tigecycline (2 mg/L for ATCC 19977 and 4 mg/L for MMX 9450) at 16 × MIC have intracellular activity against each *M. abscessus* isolate tested, with the percent reduction for omadacycline ranging from 91.74% to 97.65% compared with tigecycline ranging from 96.12% to 99.53% at similar timepoints (Figure 1). The results support the findings of Nicklas *et al.,*^17^ who reported bactericidal activity of omadacycline against *M. abscessus* in a time–kill assay and in a mouse model of pulmonary infection. Conversely, amikacin and clarithromycin demonstrated no intracellular effect across the evaluated concentrations and timepoints. These results for amikacin and tigecycline are in agreement with the data of Molina-Torres *et al*.,^11^ who investigated the intracellular activity of amikacin, clarithromycin and tigecycline against *M. abscessus* in human macrophages.

In a Phase 1 pharmacokinetic study of healthy subjects administered the FDA-approved IV dose (100 mg twice on Day 1 followed by 100 mg once daily, the exposure of which matches the FDA-approved 300 mg oral dose), omadacycline was demonstrated to have a large volume of distribution and penetrated lung tissues, including epithelial lining fluid (ELF) and alveolar macrophages, with the observed steady-state concentration of omadacycline 25.79-fold higher in alveolar cells than in plasma, and 1.47-fold higher in ELF than in plasma.^7,18^ Based on the data presented here, the concentrations of omadacycline that demonstrated intracellular activity (2 and 8 mg/L, 16 ×  the MIC for each strain) are expected to be covered by the expected human alveolar cell concentration (∼11 ± 3.72 µg/mL at 24 h), suggesting that omadacycline would be efficacious against intracellular *M. abscessus* in lung tissues.^18^ Omadacycline may be better tolerated than tigecycline due to reduced nausea—2.4% versus 47.6%, respectively—and preferred over tigecycline as it is administered once daily and can be given intravenously or orally.^7,18^ Although no new antibiotics were added to the most recently published clinical practice guidelines for the treatment of NTM pulmonary disease in 2020, recent publications have recommended oral omadacycline as a preferred initial treatment for *M. abscessus* pulmonary infections.^19,20^ However, it remains to be seen if omadacycline treatment will lead to better outcomes compared with current regimens.^20^ Presently, a Phase 2 study evaluating oral omadacycline versus placebo in adults with NTM pulmonary disease caused by *M. abscessus* is underway (NCT04922554).

In conclusion, we found that omadacycline and tigecycline demonstrated similar intracellular activities against both *M. abscessus* isolates at 16 ×  the MIC, whereas amikacin and clarithromycin displayed no intracellular activity. Our results suggest that omadacycline is a potential new agent for the treatment of *M. abscessus* infection and further studies are warranted investigating the efficacy of this therapeutic in patients with NTM pulmonary disease.

## Supplementary Material

dlad104_Supplementary_Data

## Supplementary data

Table S1 is available as Supplementary data at *JAC-AMR* Online.
