# Supplementary material for: Evaluation of omadacycline against intracellular Mycobacterium abscessus in an infection model in human macrophages
Source: JAC Antimicrob Resist. 2023 Sep 15;5(5):dlad104. doi: 10.1093/jacamr/dlad104 (PMC10502775; doi:10.1093/jacamr/dlad104)
Supplement: dlad104_Supplementary_Data [file dlad104_supplementary_data.pdf]

Organism	MIC mg/L			
	Omadacycline	Tigecycline	Amikacin	Clarithromycin
M. perigrinum ATCC 700686	0.25	0.06 (0.03-0.25)[1]	1 (≤1-4)	0.25 (≤0.06-0.5)
M. abscessus ATCC 19977	0.12	0.12	8	2
M. abscessus MMX 9450	0.5	0.25	8	2
S. aureus ATCC 29213	0.25 (0.12-1)	0.12 (0.03-0.25)	4 (1-4)	0.5 (0.12-0.5)

[1]CLSI acceptable QC value range shown in parentheses where available

Supplemental Table 1. MIC values of test agent and comparators against *M. abscessus* and QC isolates. MIC values were determined by broth microdilution following recommendations from CLSI. MIC values were used for determination of test concentrations during the intracellular time-kill assay.
